# Magnetic Properties of Cluster Glassy Ni/NiO Core–Shell Nanoparticles: an Investigation of Their Static and Dynamic Magnetization

**DOI:** 10.1186/s11671-015-0925-0

**Published:** 2015-05-28

**Authors:** Jhong-Yi Ji, Po-Hsun Shih, Ting-Shan Chan, Yuan-Ron Ma, Sheng Yun Wu

**Affiliations:** Department of Physics, National Dong Hwa University, Hualien, 97401 Taiwan; National Synchrotron Radiation Research Center, Hsinchu, 30076 Taiwan

**Keywords:** Nanoparticles, Magnetic anisotropy, Magnetization

## Abstract

We review the phenomenology of the exchange bias and its related effects in core–shell nanocrystals. The static and dynamic properties of the magnetization for ferromagnetic Ni-core and antiferromagnetic NiO-shell cluster glassy nanoparticles are examined, along with the pinning–depinning process, through the measurement of the conventional exchange bias, and associated with different cooling fields and particle sizes. Two significant indexes for the dipolar interaction *n* and multi-anisotropic barrier *β* derived from the dynamic magnetization are proposed, which provide a unified picture of the exchange bias mechanism and insight into the influence of the cooling field.

**Electronic supplementary material:**

The online version of this article (doi:10.1186/s11671-015-0925-0) contains supplementary material, which is available to authorized users.

## Background

The exchange coupling at the interface of nanosized core–shell nanoparticles, comprised of ferromagnetic metallic cores and antiferromagnetic oxide-metal shells, is of great scientific interest. With a view toward developing new approaches, the large coercivity [1, 2], hysteresis loop shift [3], and reverse hysteresis (proteresis) [4], namely the phenomenon referred to as the “exchange bias” that governs the evolution of the magnetic moment and exchange mechanism, are examined [5]. Recently, it has been found that the magnetic behavior at the interface between the cores of Ni (FM) nanoparticles with NiO (AFM) shells of various sizes can be altered [6]. The enhanced coercivity and inter-magnetic coupling that occurs as a consequence of the spin flipping [7] or spin rotation [8] of the applied magnetic field is favorable to magnetization stability. Skumryev et al. [9] examined 4-nm Co/CoO core–shell particles embedded in a paramagnetic (Al_2_O_3_) matrix. They demonstrated the existence of an exchange interaction between FM nanoparticles with an AFM matrix, suggestive of a promising way to stabilize small ferromagnetic particles against thermal fluctuations. Recently, researchers exploring the evolution of the exchange bias have been primarily concerned with how it is possible to enhance the exchange bias in a nanosized system. In a recent systematic investigation of the finite size effect [10–12], Bianco et al. observed the conventional exchange bias *H*_EB_ (CEB) in a nanogranular Ni/NiO system given various Ni weight fractions [13]. They found that with a modulation of the Ni weight fractions from 4 to 69 % but a constant NiO weight fraction (~17–19 %), there is a change in CEB at *T* = 5 K under a cooling field *H*_FC_ = 20 kOe. The maximum values of *H*_EB_ = 600 Oe and coercivity *H*_C_ = 990 Oe are obtained when the Ni weight fraction is 15 %. When the Ni weight fraction is greater than 15 %, the CEB effect tends to vanish as the size of the Ni nanoparticles increases. This is due to the corresponding decrease in the surface to volume ratio. A transition from a ferromagnetic multi-domain to a single-domain state is observed in NiO/Ni nanowires [10], which decreases with the width of the wire, concluding in the vanishing of the exchange bias when the antiferromagnet becomes a single domain.

In order to study the exchange bias of an ensemble of nanocrystals, further determination of not only the intrinsic exchange inter-coupling of the core–shell nanocrystals but also the strength of the inter-particle interactions is required. If the nanocrystals are in close proximity, for example, as in an FM/AFM or inverted AFM/FM core–shell nanocrystal system [14], they will interact magnetically via two mechanisms: the inducement of magnetic exchange across particle boundaries between particles in contact or dipolar interaction (DI) between the particles. The exchange inter-particle interaction can give rise to the anomalous spin rotation of the agglomerated particles [15]. While a fair understanding of the mechanism underlying the field cooling magnetization process and enhanced exchange bias has been achieved in the case of diluted nanocrystals, many experimental results for interacting assemblies remain unexplained. To analyze and eventually understand the experimental results obtained for the static and dynamic properties of interacting or noninteracting nanoparticles, remanent magnetization measurements were used to investigate the enhancement of the exchange bias in core–shell nanocrystals in this work. We studied the size dependence of the conventional exchange bias and anisotropic energy between the Ni-core and NiO-shell nanoparticles. The shell grew at the expense of the Ni-core, forming a passivating layer of NiO, as confirmed and estimated by the X-ray absorption near-edge structure (XANES) measurements. The conventional exchange bias (CEB) effect observed in the system after the process of field cooling through the Neel temperature was attributed to FM unidirectional anisotropy that formed at the interface between the FM-core and AFM-shell. Cooling field mapping for the study of the dipole–dipole interaction was performed by measuring the remanent magnetization at 2 K, revealing a weak dependence of the dipolar interaction on the cooling field which gives rise to the multiple anisotropic barriers observed during the cluster glassy behavior.

## Methods

The Ni/NiO core–shell nanoparticles used in the present study were fabricated by employing the thermal evaporation method. The evaporation of high-purity nickel ingots (99.99 %) mounted in a tungsten boat was initiated by first heating with a 90 A/220 V power supply. The size of the resultant Ni nanoparticles was controlled by varying the pressure in the range of 0.1–3 Torr. The flow rate of argon gas was kept constant at 10 sccm (sccm denotes cubic centimeters per minute at STP). The nanoparticles were collected on quartz plates (1 × 1 cm) maintained at 110 K, which were mounted about 12 cm above the heating source. The resultant samples were scraped from the quartz plates, appearing to be in the form of a dried powder made up of a macroscopic amount of individual Ni nanoparticles. The oxidation of nickel in the air or under humid conditions is a well-known phenomenon, but the reaction proceeds very slowly, because of the deactivation of the surface due to the formation of a thin oxide layer. In this study, an argon and oxygen gas mixture ratio of Ar/O_2_ = 5 was used to encourage oxidization of the surface of the nanoparticles in a quartz tube which was then heated at *T* = 100 °C for 1 h. The reaction at the Ni surface was terminated by the formation of a few nanometer-thick layers of nickel oxide (crystalline or amorphous phase), leading to the formation of Ni/NiO core–shell nanoparticles. Details of the shape and morphology of the prepared nanocrystals were characterized using field-emission scanning electron microscopy (FESEM; JEOL JSM-6500 F).

## Results and Discussion

### Morphological analysis and sample characterization

**Fig. 1:**
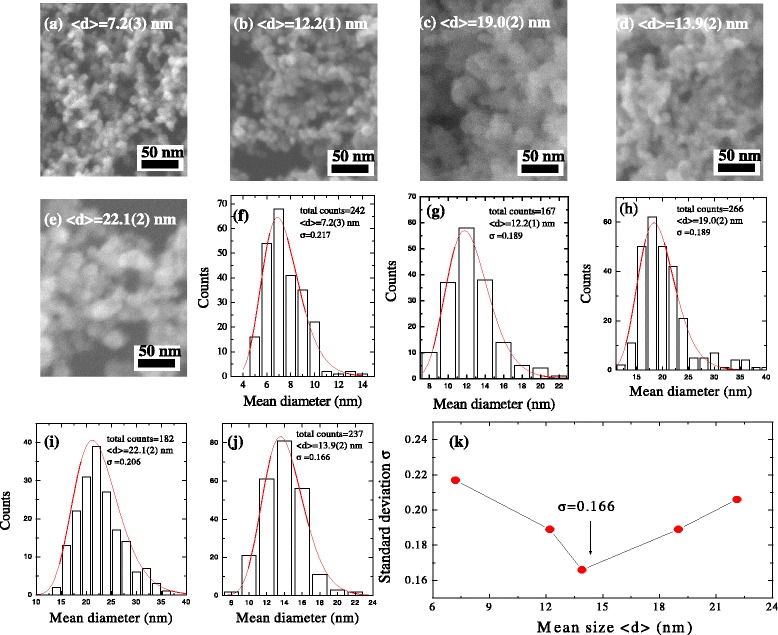
**a**–**e** The SEM images show the surface morphology of core–shell nanoparticles with various sizes. **f**–**j** The distributions of the mean size < *d* > of the Ni/NiO nanoparticles are quite asymmetric, assuming a log-normal distribution function. The mean size < *d*>, as determined from the SEM images and described by the fit to the log-normal function, were approximately 7.2(3), 12.2(1), 13.9(2), 19.0(2), and 22.1(2) nm, respectively. **k** The mean size dependence of the standard deviation *σ*. A minimum value of *σ* = 0.166 was obtained in < *d* > = 13.9(2) nm sample, revealing a narrow size distribution and offering an expected homogeneous size distribution

The SEM images in Fig. 1a–e show the surface morphology of Ni/NiO core–shell nanoparticles with various sizes. It is clearly evident that the diameters of the nanoparticles ranged from 7.2(3) to 22.1(2) nm, and the nanoparticles were quite asymmetrical, which can be described using a log-normal distribution function. The log-normal distribution is defined as follows: $f(d)=\frac{1}{d\sigma \sqrt{2\pi }} \exp \left(-\frac{{\left( \ln d- \ln <d>\right)}^2}{2{\sigma}^2}\right)$, where < *d* > is the mean value and *σ* is the standard deviation of the function. The corresponding results of the size distributions are shown in Fig. 1f–j. The values of the mean size < *d*>, as determined from the SEM images and described by the fit to the log-normal function, were approximately 7.2(3), 12.2(1), 13.9(2), 19.0(2), and 22.1(2) nm, respectively. The value of the standard deviation *σ* of the fitted function was less than 0.22 for all samples with the distribution confined to a limited range. It is worth noting that a minimum value of *σ* = 0.166 was obtained in the < *d* > = 13.9(2) nm sample, revealing a narrow size distribution and offering an expectedly homogeneous magnetic moment distribution, as shown in Fig. 1k. We find that the modulation of the mean size follows a decreasing trend with increasing the argon pressure, as shown in Additional file 1: Figure S1. Thus, the argon pressure in the thermal evaporation system can effectively control the mean sizes of resultant samples, supplying a series of mean sized samples in the present study. A summary of the parameters for argon pressure, mean diameter < *d* > and standard deviation *s* is shown in Additional file 1: Table S1). Therefore from the morphological analysis, it can be seen that uniformly distributed and well-separated spherical Ni/NiO core–shell nanoparticles can easily be synthesized by the thermal evaporation method without the use of any capping agent. It is worth noting that with the presence of a large argon pressure, spontaneous oxidation can be drastically accelerated, resulting in a large standard deviation obtained in the SEM images. The resultant nickel-based phases can be either nickel oxide or nickel-vacancy oxide phase which is the dominant product of oxidation. The nickel surface can be terminated by a few mono-layers of nickel oxide product, leading to the formation of Ni/NiO core–shell nanoparticles.

**Fig. 2:**
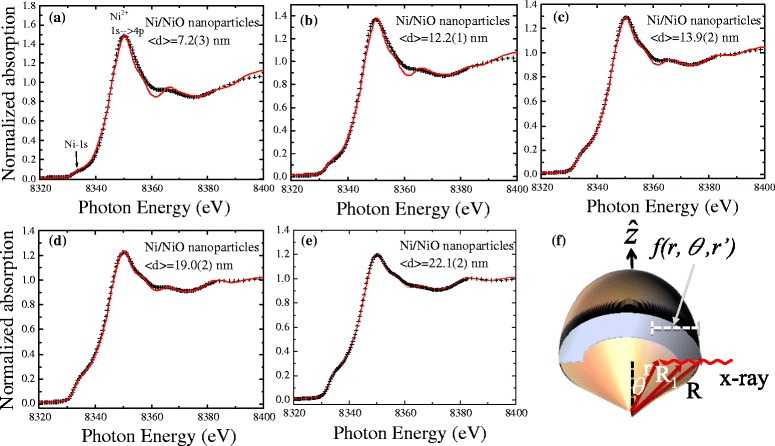
**a**–**e** X-ray absorption spectra of Ni/NiO core–shell nanoparticles. The experimental spectra and fitted curve are indicated by the *symbols* and the *red solid lines*, respectively. **f** Schematic illustration of Ni/NiO core–shell nanoparticles bias for the X-ray absorption

**Fig. 3:**
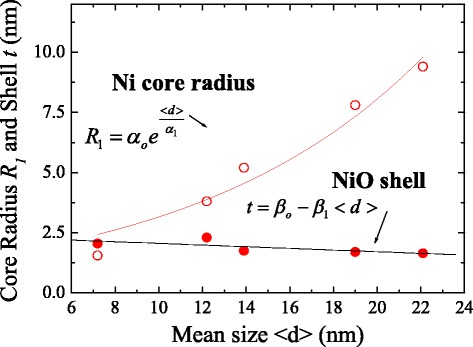
Plot of the mean size < *d* > dependence of the obtained radius *R*_1_ and shell thickness *t*. The *solid curves* indicate the fits of the data to the theoretical curves

The additional shell thickness of NiO can be detected through XANES spectroscopy which can be used to accurately examine the geometric structures, in particular, to investigate the ratio of the core to the shell. XANES spectroscopy of the Ni K_1_-edge was performed using the 6 m-HSGM beamline at the National Synchrotron Radiation Research Center in Hsinchu, Taiwan. The prepared sample was then mounted on a 3 M tape, forming a thin nanopowder. The recorded spectra were corrected for the energy-dependent incident photon intensity as well as for the self-absorption effects and normalized to the tabulated standard absorption cross sections in the energy range of 8000–8400 eV for the Ni K-edge. The observed spectra of the photon energy dependence of the intensity of Ni/NiO core–shell nanoparticles are shown in Fig. 2a–e. The main peak of Ni-1s at 8335 eV reveals the presence of a Ni phase [16]. The main peak is accompanied by a second peak at 8350 eV related to the NiO phase, which can be regarded as characteristic of Ni and Ni^2+^ (1s → 4p) [17–19], respectively. Based on earlier reports of a spherical system, a model of a single core–shell nanoparticle with an inner Ni-core coated by a thin NiO surface layer will fit the results, as shown in Fig. 2f. The intensity of the photoelectrons from the surface to the core can be described using spherical polar coordinates [18, 20] and expressed as ${I}_{\mathrm{NiO}}={I}_{\mathrm{o}}{\displaystyle {\int}_0^{\pi }{\displaystyle {\int}_{R_1}^R \exp \left(\frac{-f\left(r,\theta, r^{\prime}\right)}{\lambda_{\mathrm{NiO}}}\right)}rdr \sin \theta d\theta }$ and ${I}_{\mathrm{Ni}}={I}_o{\displaystyle {\int}_0^{\pi }{\displaystyle {\int}_0^{R_1} \exp \left(\frac{-f\left(r,\theta, r^{\prime}\right)}{\lambda_{\mathrm{Ni}}}\right)}rdr \sin \theta d\theta }$ intensity, respectively, where *I*_0_ is an instrumental constant; $R=\frac{<d>}{2}$ is the radius of the nanoparticles; and *R*_1_ is a fitting parameter for the radius of the core. The penetrating factor *f*(*r*,*θ*,*r*’) is $f\left(r,\theta, r\hbox{'}\right)=r \sin \theta -\sqrt{r{\hbox{'}}^2-{r}^2{ \cos}^2\theta }$. The mean free paths of *λ*_Ni_ and *λ*_NiO_ used in the simulation are 7.86 and 8.01 nm [21], respectively. The fitting parameter for the inner radius *R*_1_ was allowed to vary simultaneously, and refining processes were carried out until the results corresponded to the observed intensity ratio of *I*_NiO_/*I*_Ni_. It follows that the instrumental resolution function can be well converted to the observed spectrum by Gaussian functions. The ratio between the intensities of the NiO and Ni is thus determined by *I*_NiO_/*I*_Ni_, and the thickness of NiO is also obtained. The refined parameters as calculated from the model are listed in Table 1. The mean size < *d* > dependence of the obtained core radius *R*_1_ and shell thickness *t* = *R* − *R*_1_ is shown in Fig. 3. The *solid curves* indicate the fits of the data to the theoretical curves for an exponential growth and linear decay function, namely, ${R}_1={\alpha}_{\mathrm{o}}{e}^{\frac{<d>}{\alpha_1}},\kern0.24em t={\beta}_{\mathrm{o}}-{\beta}_1<d>$, where *α*_ο_ = 1.2(3) nm, *α*_1_ = 10(1) nm, *β*_o_ = 2.4(3) nm, and *β*_1_ = 0.03(1) nm and represent the initial constants and the fitted parameters, respectively. These results suggest that the NiO layer appears to be essentially a thin layer of 1.65 to 2.05 nm and the Ni-core radius *R*_1_ increases with increasing mean size. Thus, we successfully modulate a series of Ni-core coated with a constant NiO-shell thickness in the Ni/NiO core–shell nanoparticle system. This tuning of the core sizes alters the strength of the ferromagnetic moment and thereby changes the core–shell exchange interactions and intra-coupling in between nanoparticles, hinting at a possible process from multi-domain to single-domain effects for use in investigating the exchange bias.

**Table 1:** Summary of the simulated XANES results for Ni/NiO core–shell nanoparticles

<*d* > (nm)	*I*_Ni_	*I*_NiO_	Observed *I*_NiO_/*I*_Ni_	Simulated *I*_NiO_/*I*_Ni_	Ni-core radius *R*_1_ (nm)	NiO-shell thickness *t* (nm)
7.2(3)	0.3	4.03	13.43	13.44	1.55	2.05
12.2(1)	0.81	3.31	4.09	4.09	3.8	2.3
13.9(2)	1.38	2.57	1.86	1.87	5.2	1.75
19.0(2)	1.8	2.21	1.23	1.22	7.8	1.7
22.1(2)	1.7	1.72	1.01	1.02	9.4	1.65

### Temperature dependence of the FC and ZFC magnetization

**Fig. 4:**
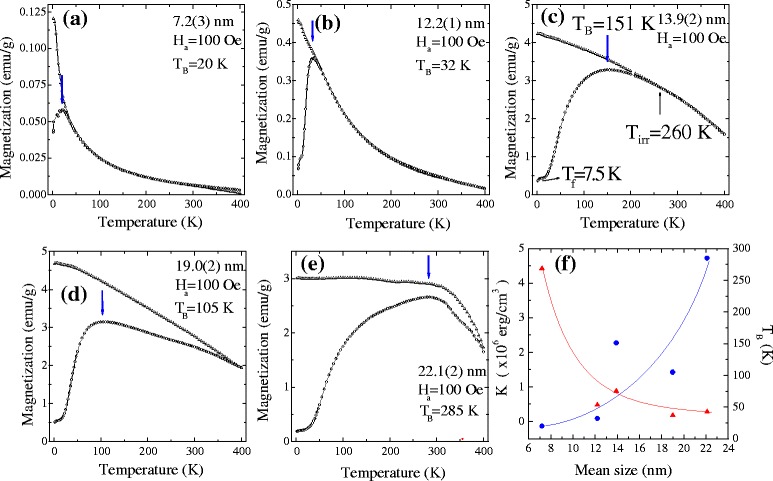
**a**–**e** ZFC and FC magnetization *M*(*T*) as a function of temperature measured at an applied magnetic field of *H*_a_ = 100 Oe. **f** Mean size dependence of the blocking temperature and magneto-crystalline anisotropy. The *solid curves* serve as a guide for the eye

The magnetic properties were measured from 2 to 300 K for all samples using a superconducting quantum interference device (SQUID) magnetometer (Quantum Design, VSM). The powdered sample was then packed into a thin nonmagnetic cylindrical holder. The temperature-dependent magnetization of the Ni/NiO core–shell nanoparticles at various sizes was measured during zero field cooling (ZFC) and field cooling (FC) with a 100 Oe field ranging from 2 to 300 K. As shown in Fig. 4a–e, the ZFC magnetization shows a pronounced broad peak for all samples while FC magnetization increases monotonically with decreasing temperatures below *T*_B_. Above *T*_B_, superparamagnetic signals dominate at higher temperature, where the magnetization decreases linearly with increasing temperature. No noticeable differences in the magnetization curves were found between the measurements made with a field-increasing loop and a field-decreasing loop. This is because the thermal excitation overcomes an energy barrier, giving rise to superparamagnetic or paramagnetic properties [5]. This result indicates that the ferromagnetic behavior [3] is mainly contributed by the Ni-core component and the moments of the cores are blocked below the blocking temperature. At lower temperatures, there is a small peak in the magnetization of the ZFC curve visible at around 7.5 K (called the freezing temperature, *T*_f_) (Fig. 4c) which can be assigned to the collective freezing of uncompensated surface NiO moments. A similar type of collective freezing of magnetic moments of NiO nanoparticles at low-temperature regions has been reported by Bisht et al. [22] However, an anomaly peak around 7 K was observed for the 7.2(3)-nm nanoparticles, as shown in Fig. 4a, due to the collective freezing of uncompensated surface NiO moments as reported by Mandal et al. [23] As the mean size of the sample increases to 22.1(2) nm, the blocking temperature increases to *T*_B_ = 285 K and the peak of the *M*_ZFC_ curve broadens due to the anisotropic distribution of the Ni nanoparticles [13, 24], as shown in Fig. 4e. The anisotropic distribution originates from the size distribution of the nanoparticles, which can be determined from the standard deviation *σ* in Fig. 1k. The increase in the standard deviation from *σ* = 0.166 to 0.217 with increases in size from 13.9(2) to 22.1(2) nm also provides us with verification of the broadening anisotropic distribution of the nanoparticles. In the case of < *d* > = 22.1(2) nm Ni/NiO core–shell nanoparticles, the FC magnetization continues to increase with decreasing temperature but with a tendency toward saturation below *T*_B_. These results can be explained by taking into account the influence of the interface anisotropy through exchange coupling triggered by applied magnetic fields, which can tune the FC magnetization of the core–shell system. Roy et al. [25] reported that the NiO shell can act as a pinning shell, pinning the core spins near the interface of the Ni inner shell and NiO outer shell via exchange interactions, preventing the core spin from rotating freely with the applied magnetic field and leading to the observed saturated FC magnetization below *T*_B_.

As shown in Fig. 4a–e, a clear bifurcation between the ZFC and FC curves is visible above the blocking temperature *T*_B_ with the curves beginning to merge at an irreversible temperature *T*_irr_. The irreversibility shown is strongly dependent on the magnitude of the applied magnetic field and is presumably associated with a slow relaxation process for an assembly of interacting nanoparticles, as discussed in a previous report. [26] These characteristics can be attributed to a dipole–dipole interaction. In a mono-dispersed system, the dipole-dipole interaction between nanoparticles can be ignored in comparison with the anisotropy energy but could be of the same order as the particle anisotropy energy in a dense system. Figure 4f illustrates the size dependence of the blocking temperature *T*_B_ obtained from *M*(*T*) measurements, which is shown to be consistent with particle size. A significant increase in the mean size < *d* > for Ni/NiO core–shell nanoparticles can be treated as due to an increase in the intra-coupling between the nanoparticles, so that a higher blocking temperature is observed. In general, the blocking temperature corresponds to an effective anisotropic energy of *K*<*V*>/25*k*_B_, where < *V* > is the ferromagnetic Ni-core mean volume. The effective anisotropy comprises several intrinsic factors such as volume, surface, shape, exchange and magneto-crystalline anisotropies. The size dependence of the calculated *K* obtained is shown on the left side of Fig. 4f. It shows the opposite behavior compared to the blocking temperature due to a reduction of particle mean volumes at lower sizes. This anomalous enhancement of the effective anisotropy indicates that the maximum in the ZFC magnetization does not correspond to the typical blocking temperature of the noninteracting particles, where inter-particle interaction, including dipole–dipole and exchange, plays a significant role in the temperature dependence of the magnetization. An enhancement of the effective magneto-crystalline nanoscale anisotropy is also observed, such as *K*_eff_ = 1.46 × 10^6^ erg/cm^3^ in < *d* > = 11 nm and *K*_eff_ = 7.03 × 10^6^ erg/cm^3^ in < *d* > = 3.71 nm, for Ni nanoparticles fabricated by the chemical reduction synthesis method [27]. Similar behaviors are also observed in Co nanoparticles [28]. Batlle et al. suggested that the enhancement of the effective magneto-crystalline anisotropy originates from the surface spin arrangement of ferromagnetic particles with a surface anisotropy.

### Size effect on the conventional exchange bias

**Fig. 5:**
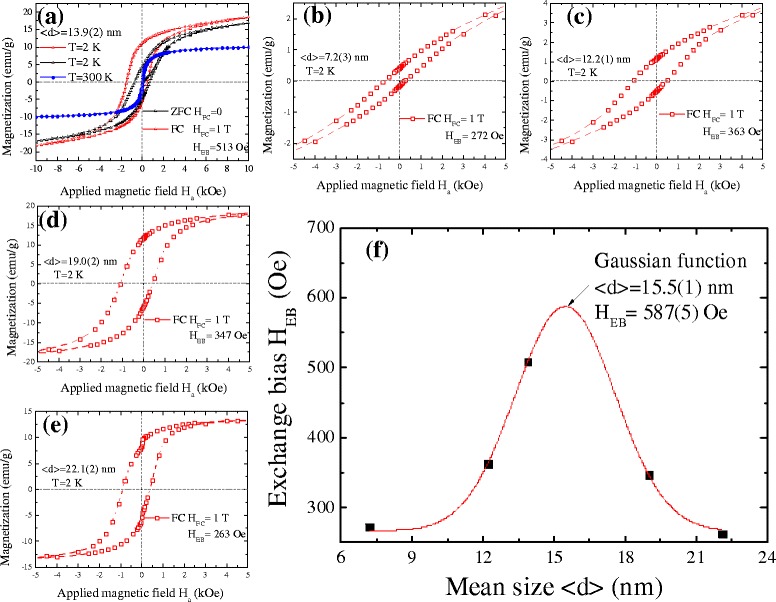
**a**–**e** Hysteresis loops measured with field cooling *H*_FC_ = 10 kOe taken at 2 K with various sizes. **f** Mean size dependence of *H*_EB_ was deduced from the FC hysteresis loops

The conventional exchange bias (CEB) occurs when a ferromagnet in direct contact with an antiferromagnet is put through the FC isothermal hysteresis loop measurement. The sample was first cooled down from 300 K to the desired temperature in an applied field of *H*_FC_ = 10 kOe. As it stabilized at the desired temperature, the hysteresis loop measurement began by setting the magnetic field to *H* = 10 kOe. Here, FC isothermal hysteresis loop measurement was carried out at 2 K. The shift of the FC loop toward the negative field, known as the CEB, can be quantified through the exchange field parameter *H*_EB_ = −(*H*_C1_ + *H*_C2_)/2, where *H*_C1_ and *H*_C2_ are the left and right coercivity, respectively. The coercivity in the FC loop is defined as *H*_C_ = (*H*_C2_ − *H*_C1_)/2. Additional file 1: Figure S2 shows an example of bulk Ni for comparison. The curve of the magnetization reaches saturation at *H*_*a*_ = 5 kOe as shown in the FC (*hollow circles*) and ZFC (*solid squares*) measurements. We observe that the FC and ZFC curves merge together, and the center of the curves corresponds to the axes of the applied magnetic field and magnetization. No CEB or coercivity was observed in the Ni bulk system, indicating the importance of the inter-coupling between Ni and NiO on CEB for the core–shell system. Figure 5a shows the hysteresis loops *M*(*H*) for Ni/NO core–shell nanoparticles with a mean diameter of 13.9(2) nm taken at *T* = 2 and 300 K (zero field cooling process). They exhibit a saturated magnetization value of 9.95 emu/g at 300 K. There are no significant differences in the magnetization measurements between the field-increasing and the field-decreasing loops found above the blocking temperature, which is consistent with the superparamagnetic behavior [29]. It can be seen that the influence of temperature on the saturation magnetization originates from the superparamagnetic characteristics in the Ni/NiO core–shell nanoparticles. The temperature is higher than the blocking temperature *T*_B_, as shown in Fig. 4c. Figure 5b–e shows a series of mean size-dependent FC hysteresis loops for the Ni/NiO core–shell nanoparticle system. There is an obvious shift in the FC hysteresis loops toward the negative field. The CEB value was estimated giving *H*_EB_ = 272, 363, 513, 347, and 263 Oe with increases in the mean size from 7.2(3) to 22.1(2) nm. The values of *H*_EB_ at various mean sizes are shown in Fig. 5f. They can be described by a Gaussian function, giving a center peak at < *d* > = 15.5(1) nm. The significant maximum conventional exchange bias found for 15.5(1) nm Ni/NiO core–shell nanoparticles was *H*_EB_ = 587(5) Oe and decreased as size increased. The abnormally enhanced exchange bias *H*_EB_ taken at *T* = 2 K may be related to the following two aspects: (i) It can be explained by taking into account the influence of the particle sizes and interface anisotropy that can tune the magnetic properties of the Ni/NiO nanoparticles through exchange coupling [30]. Unlike the usual exchange bias system, exchange inter-coupling at the Co/CoO core–shell nanocrystals [9] gives rise to a pronounced shift of the hysteresis loop along the field axis. Moreover, the size effect of CEB was also observed in the nanogranular Ni/NiO system [13] and the MnO/Mn_3_O_4_ core–shell particle system [31], with a difference in the maximum and minimum CEB values of about 550 and 2000 Oe, respectively. This result indicates the possibility of size-tuned CEB effects in the core–shell system. (ii) The shift of the FC hysteresis loops is probably related to the magnetic behavior of the frustrated AFM domains at the shell which can be regarded as a two-dimensional diluted AFM in a field (DAFF) recently reported by Benitez et al. [32] This DAFF shell can be examined by measuring magnetic field-dependent thermoremanent magnetization. Clearly, the mechanism of the enhanced exchange bias observed in core–shell nanoparticles needs further investigation.

**Fig. 6:**
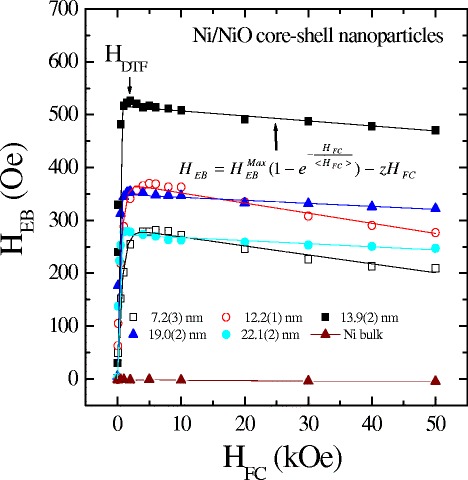
Cooling field dependence of exchange bias *H*_EB_ measured at 2 K. A collective depinning threshold field was observed and obtained at the maximum exchange bias *H*_EB_

**Fig. 7:**
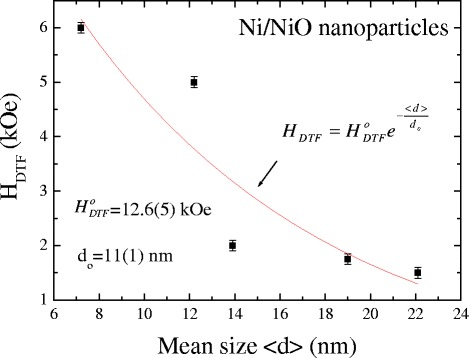
Size dependence of the collective depinning threshold field at 2 K. The *solid curve* indicates the fit of the data to the theoretical curve

In order to relate the cooling field to the exchange bias phenomena, we have to measure the exchange bias at various cooling fields. The exchange bias phenomena are expected to be dependent on the strength of the cooling field *H*_FC_, as discussed in previous studies. We investigated the cooling field dependence of the pinning–depinning processes on the obtained exchange bias of asymmetric hysteric loops in the core–shell system. Figure 6 shows the cooling field dependence of the exchange bias field *H*_EB_ for various sizes at 2 K. The negative exchange bias here is presented in absolute values. A steep increase of exchange bias was found at lower cooling field regions with increased cooling fields due to the enhancement of the core spin alignment during freezing, which attained a maximum value and then decreased. The cooling field *H*_FC_ at the maximum of the exchange bias can be considered as an effective depinning threshold field *H*_DTF_ at which the magnetic interaction is overcome by the Zeeman coupling. In general, the exchange bias field is usually thought of as the balance between the Zeeman energy of the ferromagnetic core and the surface energy due to the exchange interaction at the interface. When the cooling field is smaller than the value of *H*_DTF_, the Zeeman coupling is not strong enough to compete with the exchange inter-coupling interaction and can then produce a negative exchange bias field. The partly frozen spins in the cluster-glass regions (as discussed in Time Dependence of Magnetization) are collinear to the field direction which can cause the appearance of uncompensated spins and the exchange bias effect. The increase of the cooling field converts more and more cluster-glass regions into such frozen regions, and consequently, the exchange bias field increases with increasing cooling field. When the cooling field is larger than the value of *H*_DTF_, due to the increasing of Zeeman coupling, a weaker pinning of the ferromagnetic moment is exerted on the cluster glass, resulting in a decrease of exchange bias field with further increases of the cooling field, while the coercivity remains constant (see Additional file 1: Figure S3). When the field is large enough to destroy the cluster-glass regions frozen collinearly with the external field, the exchange bias effect diminishes again. The curve can be described by an exponential function, namely ${H}_{\mathrm{EB}}={H}_{\mathrm{EB}}^{\mathrm{Max}}\left(1-{e}^{-\frac{H_{\mathrm{FC}}}{<{H}_{\mathrm{FC}}>}}\right)-z{H}_{\mathrm{FC}}$, where ${H}_{\mathrm{EB}}^{\mathrm{Max}}$ is the CEB maximum; <*H*_FC_ > represents a fitted parameter; *z* represents the ratio of the influence of the Zeeman factor on the exchange bias; and the corresponding parameters are presented in Table 2. The mean size < *d* > dependence of the obtained effective depinning threshold field shown in Fig. 7 reveals a decrease with increasing particle size. The *solid curve* indicates the fit of the data to the theoretical curve for an exponential decay function, namely ${H}_{\mathrm{DTF}}={H}_{\mathrm{DTF}}^{\mathrm{o}}{e}^{-\frac{<d>}{d_{\mathrm{o}}}}$, where ${H}_{\mathrm{DTF}}^{\mathrm{o}}$ = 12.6(5) kOe and *d*_o_ = 11(1) represents the initial constant and the fitted parameters, respectively. This result shows that the effective depinning threshold field is controlled by the particle size and cooling field.

**Table 2:** Summary of the fitting result of the cooling field dependence of exchange bias at 2 K, covering a mean size range from 7.2(3) to 22.1(2) nm, shown as an exponential function, namely, ${H}_{\mathrm{EB}}={H}_{\mathrm{EB}}^{\mathrm{Max}}\left(1-{e}^{-\frac{H_{\mathrm{FC}}}{<{H}_{\mathrm{FC}}>}}\right)-z{H}_{\mathrm{FC}}$ where ${H}_{\mathrm{EB}}^{\mathrm{Max}}$ is the maximum value of exchange bias, <*H*_FC_ > represents a fitted parameter, and *z* represents a ratio of the influence of the Zeeman factor on exchange bias

<*d* > (nm)	${H}_{\mathrm{EB}}^{\mathrm{Max}}$ (Oe)	<*H*_FC_ > (Oe)	*z* (×10^−3^)
7.2(3)	286	724	1.69
12.2(1)	371	545	1.92
13.9(2)	517	91	0.95
19.0(2)	350	152	0.57
22.1(2)	273	159	0.59

### Time dependence of magnetization

**Fig. 8:**
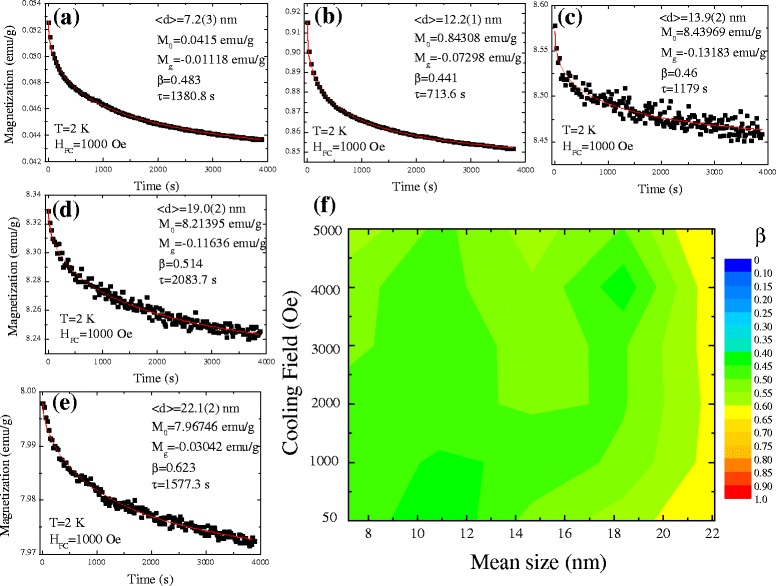
**a**–**e** Time dependence of field-cooled magnetization. The sample was cooled down to 2 K in the applied field *H*_FC_ = 1 kOe, and magnetization was recorded with time *t* in the zero field. **f** Two-dimensional plot of cooling field against the mean size < *d*>, while different colors are used to differentiate the *β* values, with *β* ~ 0 (*blue color*), *β* ~ 0.5 (*green color*), and *β* = 1 (*red color*)

In addition to the exchange inter-coupling between the Ni-core and NiO-shell, the magnetic interaction also includes the dipolar interaction between ferromagnetic nanoparticles, known as the intra-coupling interaction. How is it possible to study the influence of applied magnetic field on the dipole–dipole interaction between nanoparticles? Indeed, one of the most challenging scientific questions in core–shell nanoparticle system concerns the relaxation dynamics of an assembly of magnetic particles which is related to the decay of the recording patterns. In general, relaxation dynamics describe the decay of the remanent magnetization with time, as the applied magnetic field is turned off. There is no good definition of the functional form of the decay because the magnetic nanoparticles are poly-dispersed and sometimes, the shape and the size of the particles are well known. When investigating the influence of relaxation dynamics of cluster glassy magnetic behavior, the time-dependent magnetization was measured using the following experimental procedure. The sample was cooled down to 2 K in the applied field *H*_FC_ = 1 kOe, and magnetization was recorded with time *t* in the zero field. Figure 8a–e shows a series of mean sizes of time-dependent magnetization at 2 K. Assuming the distribution of relaxation time due to size distribution, the time-dependent magnetization was further fitted using stretched exponential functions, given as *M*(*t*) = *M*_o_ − *M*_g_ exp(−(*t*/*τ*)^*β*^), where *M*_o_ and *M*_g_ are the ferromagnetic and glassy components, respectively. The value of exponent *β* was confined in the range of 0 < *β* ≤ 1. The relaxation is included with the activation against a single anisotropic barrier for *β* = 1, revealing a paramagnetic system. As the *β* value was further decreased to 0.5, it indicates activation against multiple anisotropic barriers as observed for a cluster glassy (CG) system. The nonzero value of *β* < 0.5 stands for the distribution of multiple anisotropic barriers as observed for spin glassy (SG) systems. The *solid lines* in Fig. 8a–e indicate fits to the observed time-dependent magnetization. The obtained fitting parameters *M*_o_, *M*_g_, *τ*, and *β* are tabulated in (see Additional file 1: Table S2). The obtained fitted values of *β* are confined in the narrow range of 0.5 ± 0.02 and point toward activation against multiple anisotropy energy barriers for CG systems which is in excellent agreement with the observation from *M*(*T*) measurements. To gain further insight into the influence of cooling fields, we measure a series of cooling field-dependent magnetization as a function of time, as shown in Fig. 8f. Here, different colors are used to differentiate the *β* values, with *β* ~ 0 (*blue color*), *β* ~ 0.5 (*green color*), and *β* = 1 (*red color*). There is no significant change if the *β* value was observed under various *H*_FC_, revealing a very weak response against the cooling field. The calculated values of *β* correspond to the multiple anisotropic barriers as observed for cluster glassy nanoparticles, which possibly originated from the size distribution.

**Fig. 9:**
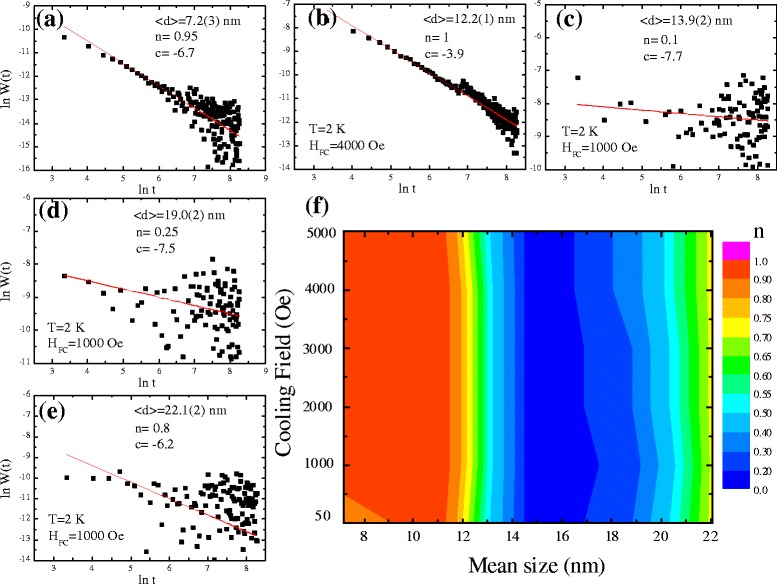
**a**–**e** Linear plot of ln *W*(*t*) vs. ln *t* at 2 K with various mean sizes. The *solid straight line* shows the fit using stretched exponential function. **f** Two-dimensional plot of cooling field against the mean size < *d*>, while different colors are used to differentiate the values of *n*. The value of *n* is crucial with *n* ~ 0 for dilute systems of mono-dispersed nanoparticles, *n* ~ 2/3 for dilute systems of poly-dispersed nanoparticles, and *n* ≥ 1 for dense systems

In addition, the ferromagnetic particles may form long chains or other types of aggregation. The dipolar interaction between the magnetic nanoparticles may also become relevant, for example, the exchange or superexchange interactions. Recently, Ulrich et al. investigated a relaxation mechanism based on the use of a Monte Carlo simulation [33] to study dipolar interactions between ferromagnetic nanoparticles. They found that the logarithmic relaxation rate *W*(*t*) can be defined as *W*(*t*) = − (*d*/*dt*)*M*(*t*). It follows a power law decay of *W*(*t*) = *At*^− *n*^ for all particle densities after some crossover time *t*_0_, for *t* ≧ *t*_0_. In the above expression, the value of *n* is crucial with *n* ~ 0 for diluted systems of mono-dispersed nanoparticles, *n* ~ 2/3 for diluted systems of poly-dispersed nanoparticles, and *n* ≥ 1 for dense systems. The validity of this theoretical model was corroborated by the relaxation dynamics for magnetic clusters [34, 35] and nanoparticles [36–38], and proved useful for identification of the strength of dipole interactions. In experiments, the sample was initially cooled down from *T* = 300 K to the lowest temperature (*T* = 2 K) under an applied magnetic field *H*_FC_. After stabilizing the temperature at 2 K, magnetization was recorded with time *t* in the zero field. Since an exponential function appears in the equation, we will use the natural logarithm. Figure 9a–e represents the ln(*t*) dependence of ln *W*(*t*) taken at 2 K under *H*_FC_ = 1 kOe over a time period of 4000 s, revealing a sharp decrease with increasing time. As shown in Fig. 9a, c, e, using ln *W*(*t*) = *c* − *n* ln(*t*), the *straight line* fits are obtained after *t*_0_ = 136 s with *n* = 0.95, 0.1, and 0.8 for < *d* > = 7.2(3), 13.9(2), and 22.1(2) nm, respectively. The obtained fitting parameters *c* and *n* are tabulated in (see Additional file 1: Table S2). The obtained *n* indicates a transformation from strong → weak → strong intra-coupling between nanoparticles with increasing size. The minimum value of *n* = 0.1 at < *d* > = 13.9(2) nm is known as a mono-dispersed interaction. Similar behaviors are obtained with the exchange bias, as illustrated by Fig. 5f. The prime source of the exchange bias is the exchange inter-coupling between the nickel spin-ordered cores and the disordered spins of nickel oxide shells. In the case of < *d* > = 13.9(2) nm, the intra-coupling between particles is weak enough to render the dipolar interaction negligible, and we can consider the obtained exchange bias to have originated from an intrinsic origin. With the increase of intra-coupling, the direct contact between the particles will become organized to produce a collective behavior that results in a reduction of exchange bias. The dipolar interaction between the FM nanoparticles was investigated by further measuring the cooling field *H*_FC_ dependent remanent magnetization, using the same experimental protocol; see Fig. 9f. Here, different colors are used to differentiate the *n* values, with *n* ~ 0 (*blue*), *n* ~ 2/3 (*green*), and *n* ≥ 1 (*pink*). It is difficult to describe the difference along the *H*_FC_-axis, which could tempt us to conclude that the remanent magnetization behaves as having a cooling field dependence. A transition from dense- to poly-dispersal is observed around < *d*_m_ > = 14–16 nm, indicating an existence of mono-dispersed nanoparticles in the region. It can be seen from the evidence that size effects will influence the strength of the dipolar interaction between the magnetic nanoparticles. If the mean size is below < *d*_m_>, the dipole–dipole interaction (*red*) belongs to a dense region, and the magnetization of the Ni/NiO core–shell nanoparticles is relatively weaker than for the other mean sizes above < *d*_m_>, as can be seen in Fig. 9f. The dense dipole interaction may originate from the contribution of the quantum size effect as discussed by Ulrich group [33]. When the mean size is above < *d*_m_>, the strength of the dipole interaction starts to increase slowly. This is due to an increase in the magnetic moment per particle, which enhances the magnetic attraction between the particles, although the magnetic attraction is still smaller than the dipole interaction in the region of dense systems, which is called a weak interaction. The above description can be used to explain the size effect of the CEB result, as shown in Fig. 5f. The enhanced exchange bias found at < *d* > = 13.9(2) nm is probably related to the mono-dispersed coupling of inter-particles which can be treated as a cluster glassy system. Strong exchange interactions can induce the phenomenon of an exchange bias at the FM and AFM or cluster-glass (CG) interfaces, which enhances the magnetic anisotropy and gives rise to the CG behavior due to the anisotropic dipolar interaction.

## Conclusions

In summary, the thermal evaporation method was used to synthesize Ni nanoparticles with Ni/NiO core–shell nanoparticles formed by the oxidation of nickel nanoparticles at room temperature. XANES and simulation analysis of the Ni/NiO core–shell nanoparticles provided evidence of the existence of a surface NiO shell (~1.65 to 2 nm) and various Ni-core diameters, forming a core–shell structure. A significant maximum value *H*_EB_ = 513 Oe was obtained in the field cooling hysteresis loops for the Ni/NiO core–shell nanoparticles (<*d* > = 13.9(2) nm). This result can be attributed to a magnetic exchange inter-coupling and can be explained by taking into account the influence of the particle size and interface anisotropy. The magnetic properties of the Ni/NiO nanoparticles can be tuned through the exchange coupling. The analysis of the relaxation of magnetization indicates a mono-dispersed inter-particle interaction for particles with < *d*_m_ > = 14–16 nm regions, giving rise to the cluster glassy magnetic behavior. Our findings show that the magnetic exchange inter-coupling, Zeeman effects, and dipolar interaction play important roles in core–shell FM/AFM systems that can then be used to describe the static and dynamic properties of magnetization.

## Additional file

Additional file 1: **Supplementary material.** A file containing three supplementary figures and two supplementary tables.
