# Supplementary material for: Magnetic Properties of Cluster Glassy Ni/NiO Core–Shell Nanoparticles: an Investigation of Their Static and Dynamic Magnetization
Source: Nanoscale Res Lett. 2015 May 28;10:243. doi: 10.1186/s11671-015-0925-0 (PMC4452478; doi:10.1186/s11671-015-0925-0)
Supplement: Supplementary file 1 — Supplementary material. A file containing three supplementary figures and two supplementary tables. [file 11671_2015_925_MOESM1_ESM.doc]

# Supplementary material

# Magnetic Properties of Cluster Glassy Ni/NiO Core-shell Nanoparticles: An Investigation of their Static and Dynamic Magnetization

Jhong-Yi Ji^1^, Po-Hsun Shih^1^,Ting Shan Chan^2^, Yuan-Ron Ma^1^, and Sheng Yun Wu^1,*^

^1^Department of Physics, National Dong Hwa University, Hualien 97401, Taiwan

^2^National Synchrotron Radiation Research Center, Hsinchu 30076, Taiwan

**Figure S1** The modulation of the mean size employing argon pressure in the thermal evaporation method.

**Figure S2**: Conventional exchange bias in the samples investigated through examination of the applied magnetic field cooling (FC) hysteresis loops at T= 2 K. In the FC process, the sample was cooled down from the room temperature to the measurement temperature T=2 K under an applied magnetic field *H_FC_*=1 T. The hysteresis loops with *H_FC_* for bulk Ni are shown in this figure.

**Figure S3** The cooling field H_FC_ dependence of the coercivity at 2 K covering a mean size range from 7.2(3) to 22.1(2) nm. Its organization is characterized by a rapid initial rate that slows down and then terminates. The curve can also be described by an exponential function, namely $H_{C}=H_{C}^{Max}(1-e^{-\frac{H_{FC}}{<H_{FC}>}})+H_{o}$, where H_0_ indicates the initial coercivity at H_FC_=0; $H_{C}^{Max}$is the maximum coercivity; and <H_FC_> is a fitted parameter. The corresponding parameters are presented in Table S1. We note that the maximum coercivity $H_{C}^{Max}$ increases to 225 Oe with an increase in the mean size to 13.9(2) nm, and then decreases to 121 Oe with a further increase in the mean size to 22.1(2) nm. The change of $H_{C}^{Max}$ originates from the influence of the size effect, with a result similar to $H_{EB}^{Max}$. When the field cooling strength is above $H_{FC}^{Max}$, the coercivity reaches the saturation point. It can be seen that the higher field cooling strength and Zeeman effect no longer affect the value of coercivity.

**Table S1** The summary of parameters for argon pressure, mean diameter <d> and standard deviation σ.

| Ar pressure (torr) | <d> (nm) | σ |
| --- | --- | --- |
| 2 | 22.1(2) | 0.217 |
| 2.5 | 19.0(2) | 0.189 |
| 3 | 13.9(2) | 0.166 |
| 3.5 | 12.2(1) | 0.189 |
| 4 | 7.2(3) | 0.206 |

**Table S2** Summary of the fitting results presented in Figures 8 and 9, respectively, for the cooling field dependence of dynamic magnetization with various sizes.

| <d> (nm) | M_0_ (emu/g) | M_g_ (emu/g) | τ (t) | β | n | c | H_FC_ (Oe) |
| --- | --- | --- | --- | --- | --- | --- | --- |
| 7.2(3) | 0.0007 | -0.0008 | 1760.821 | 0.48821 | 0.85 | -10 | 50 |
| 7.2(3) | 0.0095 | -0.0014 | 1535.248 | 0.49504 | 0.9 | -9.2 | 100 |
| 7.2(3) | 0.0415 | -0.0112 | 1380.79 | 0.48303 | 0.95 | -6.7 | 1000 |
| 7.2(3) | 0.0452 | -0.0141 | 942.9548 | 0.48865 | 0.9 | -6.7 | 2000 |
| 7.2(3) | 0.0467 | -0.0154 | 1003.817 | 0.50631 | 0.9 | -6.6 | 3000 |
| 7.2(3) | 0.0475 | -0.0162 | 1048.154 | 0.50905 | 0.9 | -6.5 | 4000 |
| 7.2(3) | 0.0486 | -0.0162 | 864.3421 | 0.5934 | 0.9 | -6.5 | 5000 |
| 7.2(3) | 0.0482 | -0.0177 | 928.0904 | 0.49856 | 0.9 | -6.5 | 10000 |
|  |  |  |  |  |  |  |  |
| <d> (nm) | M0 (emu/g) | Mg (emu/g) | τ (t) | β | n | c | HFC (Oe) |
| 12.2(1) | 0.1354 | -0.0045 | 1104.6 | 0.405 | 0.95 | -7.7 | 50 |
| 12.2(1) | 0.2855 | -0.0087 | 953.8 | 0.453 | 0.95 | -7 | 100 |
| 12.2(1) | 0.8431 | -0.0730 | 713.6 | 0.441 | 1 | -4.5 | 1000 |
| 12.2(1) | 0.9870 | -0.1084 | 1032.3 | 0.485 | 1 | -4.2 | 2000 |
| 12.2(1) | 1.0549 | -0.1273 | 1022.3 | 0.483 | 1 | -4 | 3000 |
| 12.2(1) | 1.0834 | -0.1375 | 1000.2 | 0.483 | 1 | -3.9 | 4000 |
| 12.2(1) | 1.0952 | -0.1367 | 1194.8 | 0.484 | 0.95 | -4.2 | 5000 |
| 12.2(1) | 1.1229 | -0.1572 | 1048.2 | 0.490 | 0.95 | -4.1 | 10000 |
|  |  |  |  |  |  |  |  |
| <d> (nm) | M0 (emu/g) | Mg (emu/g) | τ (t) | β | n | c | HFC (Oe) |
| 13.9(2) | 1.6329 | -0.0068 | 210.7 | 0.518 | 0.1 | -9.5 | 50 |
| 13.9(2) | 3.3770 | -0.0151 | 1213.0 | 0.523 | 0 | -10 | 100 |
| 13.9(2) | 8.4397 | -0.1318 | 1179.0 | 0.460 | 0.1 | -7.7 | 1000 |
| 13.9(2) | 9.4651 | -0.2125 | 1000.7 | 0.509 | 0.1 | -8 | 2000 |
| 13.9(2) | 9.8825 | -0.2463 | 1099.5 | 0.511 | 0.1 | -8 | 3000 |
| 13.9(2) | 10.1035 | -0.2560 | 967.7 | 0.542 | 0.1 | -8 | 4000 |
| 13.9(2) | 10.2423 | -0.2738 | 873.7 | 0.572 | 0.1 | -8 | 5000 |
| 13.9(2) | 10.4392 | -0.3127 | 1090.0 | 0.473 | 0.1 | -8 | 10000 |
| <d> (nm) | M0 (emu/g) | Mg (emu/g) | τ (t) | β | n | c | HFC (Oe) |
| 19.0(2) | 1.5140 | -0.0135 | 344.1 | 0.574 | 0.3 | -9 | 50 |
| 19.0(2) | 2.8149 | -0.0268 | 384.8 | 0.547 | 0.4 | -7.8 | 100 |
| 19.0(2) | 8.2140 | -0.1164 | 2083.7 | 0.514 | 0.25 | -7.5 | 1000 |
| 19.0(2) | 9.4350 | -0.1436 | 943.7 | 0.495 | 0.3 | -7 | 2000 |
| 19.0(2) | 9.7290 | -0.1829 | 1249.1 | 0.493 | 0.3 | -7 | 3000 |
| 19.0(2) | 9.8392 | -0.2317 | 1833.6 | 0.419 | 0.35 | -6.5 | 4000 |
| 19.0(2) | 9.9790 | -0.2264 | 1159.2 | 0.498 | 0.35 | -6.7 | 5000 |
| 19.0(2) | 10.0688 | -0.2406 | 1259.7 | 0.457 | 0.35 | -6.7 | 10000 |
|  |  |  |  |  |  |  |  |
| <d> (nm) | M0 (emu/g) | Mg (emu/g) | τ (t) | β | n | c | HFC (Oe) |
| 22.1(2) | 2.3216 | -0.0223 | 1186.2 | 0.601 | 0.8 | -6.5 | 50 |
| 22.1(2) | 3.7746 | -0.0069 | 1827.3 | 0.666 | 0.4 | -9.5 | 100 |
| 22.1(2) | 7.9675 | -0.0304 | 1577.3 | 0.623 | 0.8 | -6.2 | 1000 |
| 22.1(2) | 8.5727 | -0.0575 | 1199.8 | 0.619 | 0.8 | -5.6 | 2000 |
| 22.1(2) | 8.7276 | -0.0701 | 665.3 | 0.628 | 0.8 | -5.5 | 3000 |
| 22.1(2) | 8.7472 | -0.0713 | 851.9 | 0.643 | 0.8 | -5.5 | 4000 |
| 22.1(2) | 8.9355 | -0.1052 | 1279.5 | 0.679 | 0.8 | -5.2 | 5000 |
| 22.1(2) | 8.8632 | -0.0756 | 955.0 | 0.647 | 0.82 | -5.4 | 10000 |
